# Charlatans and Empirics, or Dentistry as a Learned Profession

**Published:** 1871-08

**Authors:** A. P. Stevens


AMERICAN JOURNAL

OF

DENTAL SCIENCE.

Vol. V. THIRD SERIES--AUGUST, 1871. No. 4.

### ARTICLE I.

### *Charlatans and Empirics, or Dentistry as a Learned Profession.*

By A. P. Stevens, D. D. S.

**Read before the Merrimac Valley Dental Association.**

* * * * * I now invite your attention to my subject for the evening, which is entitled "*Charlatans and Empirics, or Dentistry as a Learned Profession.*"

Such of you as have been subscribers to any considerable extent—and I trust all of you have—of periodical dental literature, cannot have failed to remark the frequency of articles reviewing the early struggles and rapid upward progress of the profession of dentistry. Indeed, so closely have such articles been interwoven within the pages of our representative journals,—the Cosmos, Times, Journal and Register,—for the past decade of years, that I have often thought the little army of writers so employed must be strangely enough possessed of the idea of the danger we are in, and so are engaged in the laudable occupation of "whistling aloud to bear their courage up;" or, in lieu of this, are members of a great admiration society, whose business it is to tickle their own and each other's ears every convenient occasion.

I would not be understood by these expressions to wholly or mainly deprecate this spirit of pride and self-laudation; on the contrary, possessed as I am of deep and absorbing love for my profession, I find much to respond to, and much to commend in such articles. Oftentimes when harrassed and wearied through vain efforts and humiliating failures, I have had recourse to such reading, and risen to better efforts, refreshed in mind and body, in view of successes achieved by others more laborious, or more fortunate than myself. Truly we have reason for pride, but still none the less for distrust and watchfulness. Remember, it is truism not only in political warfare, where it is best illustrated, but in the conduct of every enterprise, that excess of confidence detracts from effort, and undermines one's best chances of success. Realizing this to its fullest extent as it applies to our profession, I voluntarily take upon myself the unpopular duty of calling your attention to a few ugly truths and facts; or, in other words, instead of joining in the glad acclaim of praise, to sound the notes of alarm in our camp.

I am to speak to you of Charlatans and Empirics. Let me first give character to the words I have chosen; then make it plain to you that we have such characters in our midst; next convince you that such persons are out of character in our profession; and last, that we, as a profession, are characterless, if we do not frown them from our ranks. To all intents and purposes, the terms Charlatan, Quack and Empiric are synonymous. I give you Webster's definition of each :—

*Charlatan*—One who prates much in his own favor, and makes unwarrantable pretensions to skill; a quack; an empiric.

*Quack*—A boaster; one who pretends to skill or knowledge which he does not possess; an ignorant practitioner.

*Empiric*—An experimenter; a physician who entered on practice without a regular professional education; hence the word is used also for a quack, a charlatan.

These are Webster's words, gentlemen, and Worcester gives us others like them. I confess that as I review my own twelve years of professional struggle, and attempt to weigh in the same balance a majority of my fellow practitioners, they sound hard and harsh; that the line is drawn painfully, remorsely close, but still we must accept them. We cannot do otherwise if we would. What think you, does the word portraiture of the great lexicographer apply to any among the twelve thousand dentists in our country? To any among our own number? Let us look the question fairly in the face, with no spirit of recrimination or envy, but as honest men, who, knowing there is a spirit of Judas in our midst, are more filled with fear lest it should be found with ourselves, than desirous of charging it upon our neighbors. Do any of us prate much in our own favor? Do any of us make unwarrantable pretensions of skill? Do any of us boast, yet know how frail is the foundation beneath our feet? Do any of us arrogate to ourselves the possession of knowledge, concerning which we would not dare be questioned? If so, and I say it in no spirit of self-security and egotism, no matter in what proportion such persons stand in point of number to the great army of dentists in America, then are such quacks, charlatans, empirics, and are guilty before their profession, and the public whom they victimize, just in proportion as they are possessed of innate talent and ability, which, if developed, would raise them above the level of this stigma. Do you accuse me of harsh words now, of unkind denunciation? But remember, I am not claiming invulnerability; I am not saying, "stand by I am holier than thou." Gentlemen, I fear there are a great many charlatans and empirics in our profession. More than that I cannot resist the conviction that some of us who have felt most secure, are not without the stain of this foul blemish upon our professional garments. What is the remedy? It is a simple one, and though not without its weariness and toil, yet none the less plain, and none the less royal,—more of brain culture with that of manual dexterity; more of underlying principles before attempts at practice; more of close and searching study and less of cunning craft; more of the conviction that the dignity and honor of our profession is committed to us, rather than dependent upon the memory of the departed. Let us look backward for a moment and consider whence we are; perhaps by this means we can best determine what we are, and what we shall be.

Mr. Phillips, in his lecture upon the "Lost Arts," indirectly asserts that dental surgery flourished as a distinct branch of science in the days of the old Egyptian Empire, more than three thousand years ago.* For one, I should be glad if this belief might be fully substantiated; for I have a profound veneration for antiquity, with its hidden, mystical lore, but a still more profound regard for anything that honors my profession. Be this as it may, we have no fear of our art being esteemed wanting in years, or lacking in great names that have been identified with it. The lustre that emanates from the name of Hypocrates; of the historians, Herodotus and Pliny; the poet Horace; of Celsus and Galen; and still later, of Eustachius, Hunter, Fox, Bell, Kercker, and a score of others, falls as legitimately and gracefully upon us, as it does upon the disciples of medicine and general surgery. Add to these a Harris, a Hayden, a Townsend and a Flagg, with scores of other names of our own day, whose genius and talent have lighted up the dark way these one, two, three and four decades, and we have a wealth of research and fame that may well satisfy the most grasping miser of us all. But this is not the whole of dentistry in the past. If it has had its good men, and its great, is has also had its full share of fools and knaves. Superstition and ignorance have walked unchallenged through its ranks these many scores of years, and gathered to themselves their followers by tens of thousands. What otherwise

*In support of this assumption, we have the words of Herodotus the Greek historian, who said:—"The art of medicine is so practised in Egypt, that there is found an individual healer for each distemper. Hence the whole country is filled with healers; some take charge of the disorders of the eyes; others of those of the head; others of those of the teeth; others of those of the belly, etc."

could we expect? A crude science without boundaries or definitions, a crude unlearned fellowship, was a natural sequence. But despite the general darkness and gloom of our past, there were bold hearts and brave spirits in those days—men who could not be satisfied with the little store of knowledge bequeathed them, but who with ever longing desires stretched out their hands into the unknown future, eagerly searching after truth.

Science ever opens its store houses to him whose ceaseless importunities will not be denied. Thus little by little mystery after mystery was unfolded; truth after truth defined; principle after principle established, and a world of knowledge opened before them. But not for themselves alone were these investigations pursued; not for their gain alone, but as legacies to all coming time—for you and me. Even as the hardy pioneers of the trackless Western world pushed on their tireless march, overcoming obstacles and dispelling doubts, that advancing civilization might have unobstructed paths, so these men whom I have named, and many others equally honorable, toiled and labored in the unexplored domain of science, that the men of the present time might have a sure foundation upon which to rear a lofty superstructure—*a system of thorough dental education.* Let us glance at the means thus placed at our disposal, and see if they are adequte to the end.

Fauchard, who by many writers is termed the "father of dentistry," published in 1720 the first comprehensive treatise upon the theory and practice of our profession. The work saw two editions, and for many years stood at the very head and front of dental literature. The next work of note was by Jourdain, in 1761. After this came the immortal John Hunter's "Natural History and Diseases of the Human Teeth." These, with a publication by Robert Blake, filled up the interim to the year 1803, when Fox gave the world the result of his researches in his two famous works, which, as revised under the title of "Fox and Harris on the Human Teeth," by reason of its honorable age and sterling merit, should have a place in ever dentist's library. Leaving the works of the fathers, we turn to those of their sons, in the persons of our countrymen.

In 1822, Eleazer Parmly published the first work upon the subject of dental practice ever issued from the American press. It was a small monograph entitled "Diseases and Treatment of the Teeth." About the same time, Dr. J. F. Flagg published a similar treatise. This was followed during the next few years by various papers on dental subjects, all of which were presented to the profession through the medium of the medical journals published in New York and Philadelphia. But it was as late as 1829 before the first volume worthy of being ranked as a text book for students, was published by an American author. This was an illustrated work of 500 pages, by Samuel S. Fitch. Those who have had access to the volume—I have vainly tried to obtain it—speak of it as being very thorough and comprehensive for that period of our profession.

In 1839, Chapin A. Harris—a name at whose mention we should all bow—gave us the first edition of his "Principles and Practice," then published under another title. I presume this work, in the shape of one of its advanced editions, is possessed by all of you; if not, it should be, for every dentist in America, at least, owes him a debt of gratitude, the payment of which can only be approximated by the purchase and careful study of his two great works—the volume I have referred to, and his "Dictionary of Dental Surgery." Since the year 1840, which, from the fact of its being marked by the establishment of the first Dental College, and the first Dental Journal in the world, is looked upon as an epoch in the history of dental literature, there have arisen many able writers who have given us volume after volume, some of which bring to view the very foundation of our art, and present its many intricate paths with a masterly hand. Prominent among these are "Piggot's Dental Chemistry;" Bond's Dental Medicine;" "Taft's Operative Dentistry;" "Jacobi on Dentition;" Robertson on Extracting Teeth;"—

a valuable book by the way for students; and last, though by no means least, "Diseases and Surgery of the Mouth, Jaws and Associate parts," by James E. Garretson. May God bless him and his labors.

In 1840, the "Baltimore College of Dental Surgery" was founded—the first in America, the first in the world. In 1846, the "Ohio College" was established in Cincinnati. In 1856, the "Pennsylvania College" sprang into existence, followed in 1863 by its sister institution in the same city—the "Philadelphia Dental College." Since which time there have rapidly risen the Missouri College, in St. Louis, the New Orleans, the Harvard, and the Boston Colleges. Nine well established Dental Colleges in our land, each with its corps of earnest, indefatigable men,—men who in the main would take rank in any calling, and who esteem no time wasted, no exertion without its rich reward, that adds to the attainments and dignity of any member of our profession.

*What say you gentlemen, have we been lacking in available means wherewith to make ourselves artistic men? Scientific men? educated men? Could any one with a desire for knowledgs have failed of decent attainments within the last score of years, without shutting his eyes to this long list of advantages offered him?* I think not. Perhaps it will be as well here to glance at some of the essential qualifications this half century of constant progression imperatively demands of him who would fulfill his obligations as a dental practitioner.

I need hardly remind you that the dentist of to-day cannot, with honor, occupy the same place as did he of 1820 and 1840. Then, even though honesty and earnestness had been the characteristics of every disciple of our art, the lights that shone in upon his upward pathway were flickering, in comparison to the noonday clearness that now illumines every step. Then knowledge was wrung out by patient toil; by laborious investigations; by pains-taking records and repeated experiments. Now, the field is clear, and the accumulation of a thousand minds, in a half century of years is given us for the occupation. No faculty of Dental College; no well established curriculum of studies; no library of voluminous literature waited upon the Haydens, the Parmlys, and the Harrises of those days, but to us, all this and more is offered almost for the acceptance. Need I say that he who fails to appreciate this difference, or refuses to manfully meet the long array of obligations devolving upon him, is unworthy of his calling, and of his position, however humble it may be.

Fifty years ago, no other demand was made upon the rank and file of the one hundred dentists in America, than ingenuity, and the cunning of hand-craft sufficed to furnish. But to-day all is changed. A trade has given place to a glorious science; a divine art, and the dentist of 1870 needs as good natural endowments, with as high mental culture, and as close application to the deeply underlying principles of his profession as do the disciples of law or general medicine. Founded upon a knowledge of Metallurgy; the principles of Natural Philosophy and Mechanics; a fair acquaintance with Anatomy, Histology, Physiology, Pathology, Chemistry and Therapeutics, nothing less than an earnest and a studious nature will make him master of his calling, and give him that confidence in himself and his resources that insures the successful practitioner.

My friends, have I overdrawn the picture? Have I added one line more than is needed to make up the sum total of what should be your attainments and mine, or of what should be the qualifications of any young man entering our profession? This is an important question for our consideration. In my own mind it was answered years ago. The acquisition of my profession has not proved to me the lightsome task I once esteemed it; but though I brought to it youth, earnestness and ambition, I have ever found the gain of each year has only served to open my vision to other fields, yet to be occupied before the seal of mastery can be set. I do not propose in this paper to sound any uncertain note, or yet to take any position the nature of which can be doubted, or yet to give any false alarm.

If the observation of years, and the spirit of the letters I have read to you, are worth anything as guides, then I am justified in saying that we need a reform in our profession, and that the strong influence of public opinion, and the arm of the law should be evoked as a guard and protection. Why should members of our profession shrink from this course? Are the men in our ranks higher, and are we stronger than the professions of divinity, general medicine and jurisprudence? And yet each of these are encircled as closely as a walled city by one or the other of these influences. Dentistry alone, of all the professions, is the open field of occupation for incompetency and ignorance. Is it any wonder that quacks and charlatans are scattered all over our broad land, and that the work of devastation steadily goes on, though ten times ten thousand outraged mouths yearly cry out against us, while our profession, which in its remote and approximal relationships, takes such fast hold of human weal and woe, is degraded in so many instances to the trade of butchery, and the lowest level of hand craft?

It is is estimated that there are sixty thousand practitioners of general medicine and surgery in the United States, and that nine-tenths of these are regular graduates of medical schools, while more than a third of this number have been the recipients of a scientific and classical education. Our own special division of medical science counts upwards of eleven thousand practitioners.

Of this number there have been graduated 662 by the Baltimore College; 223 by the Ohio College; 483 by the Pennsylvania College; 153 by the Philadelphia College; 40 by the New York College; 26 by the Boston College; 30 by the Missouri College; 38 by the New Orleans College and 18 by the Harvard College; 1,673 graduates in all, or hardly more than one in seven who have evinced by their actions that there is anything in the profession of dentistry, the mastery of which requires any other preparation than the ordinary routine of office practice will readily furnish.

Is this anything to be proud of? Is this making dentistry the peer of medicine and law? I confess I cannot get up any enthusiasm over these facts, or feel that despite the many triumphs of individual members, our profession, as a whole, is making good its boastful pretentions. It does not matter to you and me what men in the past have accomplished, or what men far removed from us in point of culture and education are doing now to honor our profession, unless we take fast hold of the results of their labor, and by a thorough assimulation of them, make them our own.

There is no disguising the fact, the action of each of us in selecting dentistry for our calling in life, is a plain declaration that we esteem it the merest trade, easy of mastery by any other than a blockhead, or else that we look upon it as the full relationship of any and all the divisions of medicine and surgery, and only to be acquired and excelled in, through the close and and painstaking studentship that characterizes the successful man in any profession. I do not by this, mean to be understood as assigning College graduates to the one class, and all who are without dental diplomas to the other. By no means. To my certain knowledge there are blockheads and quacks in our profession who are, and from the very nature of their organizations, always will be quacks and blockheads, in spite of yard long parchments, while it is well known there are many bright ornaments to the dental profession in every State, whose names have never been undersigned by College faculty. All very true; but this does not absolve honest men in our profession from the obligations they are under, to it.

No man in our ranks who, unaided by extensive reading or College lectures, has worked out a successful practice, but is an object of anxious regard to his younger brethren; and well may they, and the students he is ushering into professional life, reason that if the solid rewards of labor can be thus gained, why may not they pursue the same course. This is but natural, as it also natural for the young and uninitiated in any calling to overlook the painful toil, and often times world of humiliating failures that must be met and conquered before success can be thus achieved. As evidence of an element only too rife in our midst, a student of mine was not long since told by a young practitioner that he would not give a dime for all the books upon dentistry, or a penny for its theories ; *practice was all he wanted.* This coming from an accredited and boastful young man, who cannot for the life of him give the names and authors of our common text books, and who stands avowedly aloof from such gatherings as the present, lest his shallowness should be exposed, speaks volumes at a time like this. And another of very much the same standing, though older in his practice, unblushingly assured me that it was pretty sounding, this talk about professional standing, but for his part he preferred bread and butter for his family, and should take such a course as would secure it, regardless of any "code of ethics" or opinions of others. Unfortunately these are the thoughts and determinations of many, and are like to be those of many more.

Short sighted policy that cannot see that failure to develop one's greatest power of usefulness, is the surest way to debar the highest rewards in any calling. I can safely challenge the world to show me a dental practitioner in whom sterling honesty of character is united with high professional attainments, that has not been the recipient not only of honor and regard, but also of that other reward which sweetens labor—gold.

Careful men in our ranks estimate that there are some two thousand persons in the United States calling themselves dentists who, within the last half score of years, have ridden roughshod and unwashed into our profession through the great highway of vulcanite and cheap dentures, and who to-day are not only dishonoring the name of dentistry by their glaring pretension and palpable deficiences, but cursing humanity by a wholesale forcing of their ill made wares upon an unenlightened and deluded people. These are the men who fluant their guady handbills at every street corner ; who flood house and shop with outrageous pretensions to knowledge, the first rudiments of which they bid fair never to possess ; who in their stereotyped advertisements, practically assure the public that a year or two of laboratory studentship, oftentimes only of a few months, fits them for a successful crusade against all the ills dental organs are heir to, and warrants them in a general slaughter of the innocents under cover of chloroform, ether and gas, and who can boldly *guarantee you entire satisfaction in every operation.* These are the men, found in every State of the Union, who assure you that vulcanizers are the great invention of the day, and vulcanite the greatest blessing vouchsafed fallen humanity. These are the men who making gas and ether the pivots upon which their whole practice turns, extract their twenty, thirty and even sixty teeth daily, that thereby a knowledge of the beneficient qualities of this delectable compound of *gum*, *sulphur* and *mercury* may be more widely extended, and their dear patients thus spared the "*extortionate*" demands of some hard working, conscientious practitioner, who unfortunately believes that God's wisdom and handiwork is greater than man's. These are the men who fill all sorts of cavities in all classes of teeth, with amalgam and artificiel ; gravely assuring their trusting victims that it is just as good as gold, while it costs only half as much.

But enough! I only waste words in thus attempting to delineate characters you are all familiar with. Their devilish work speaks for them, and is scattered all over our broad land. and their baleful influence presses with the weight of a mill stone upon our whole profession, daily standing as an impassable barrier before the purest and highest efforts. What shall be done? The evil is before us, and it must be met. If this class is numerous now, what will the next ten years bring us to? The same open gateway is before them ; the same class of deluded dupes stand ready to receive them.

Already these worse than charlatans and quacks swarm in upon us as did Austrian pandoras upon Great Frederick's harassed and bleeding armies, and unless we, as did Frederick, strike our blows for their dismemberment or utter annihilation, not the memory of Fox or Harris, or fame of Owen or Tomes, or scores of Dental Colleges can save us from derision and scorn. The world furnishes but few samples of reform, or great good accomplished, save through a spirit of self-sacrifice and a willingness to labor. This well trodden pathway alone is open to us now. Dentists of honor and integrity owe it to themselves that they became such in the broadest and truest signification of the term. To this end professional standing must be sought for, labored for, and a high and noble spirit of emulation fostered and encouraged, not only in themselves, but in their brethren. True dentists of 1870 owe it to themselves and their profession, that their title of doctor has been well earned and is legitimate, and not extended to them solely through the courtesy of a good natured public. Here is the pathway of honor for willing feet to walk in, and here where the spirit of self-sacrifice is needed. There is toil and self-denial in this same highway, but the weariness it brings is the sure harbinger of gathering strength, and the thrice proven prophet of coming gladness. I do not advocate dental collegiate education because I esteem this once accomplished, all has been done. By no means. This is but the beginning, and its greatest value will ever be in its moral effect, and the power it has to sharpen vision to hitherto unknown defects, and in opening unthought of fields for future occupation.

That man is barren of manhood's true ambition who can put away a busy practice for months, and betake himself to the routine of lecture and study without coming from the scene of his labor and triumph with brain and hand stimulated to higher aud better efforts. Here should be the beginning of reform. Already hundreds of earnest, thoughtful men have directed their footsteps towards the Mecca of their hopes—Dental Schools—beacon lights of our profession, stamping themselves by the act as the friends of education and advancement, and where hundreds have gone, thousands are needed. Let there be a thorough separating of the wheat from the chaff; a whole hearted coming up to duty; a dividing line drawn that shall be seen and felt, and this alarming influx of drift-wood shall be stopped forever. It needs no prophetic vision to foretell our future. The handwriting upon the wall is in no mystical character, but can be known and read of all. To the honorable man and patient toiler it tells of hope, while to tne ndolent and the knavish it speaks all too plainly of the coming time when the mask of false pretention shall be torn away by an educated people, and they shall stand forth in all their native deformity.

Already several of our States have spoken in their might, and by the enactment of just laws throw a shield of protection around abused and suffering communities, and it needs only a passing knowledge of events and principles governing our calling, to speak with certainty of the day when the same wholesome legislation shall prevail all over our broad land. May love, truth and justice speed it.

